# *Rickettsia felis* identified in two fatal cases of acute meningoencephalitis

**DOI:** 10.1371/journal.pntd.0007893

**Published:** 2020-02-18

**Authors:** Arthur H. P. Mawuntu, Edison Johar, Riane Anggraeni, Feliana Feliana, Janno B. B. Bernadus, Dodi Safari, Frilasita A. Yudhaputri, Rama Dhenni, Yora P. Dewi, Cecilia Kato, Ann M. Powers, Ronald Rosenberg, Amin Soebandrio, Khin S. A. Myint

**Affiliations:** 1 Faculty of Medicine, Sam Ratulangi University, Manado, Indonesia; 2 Emerging Virus Research Unit, Eijkman Institute for Molecular Biology, Jakarta, Indonesia; 3 Division of Vector-Borne Diseases, Centers for Disease Control and Prevention, Fort Collins, Colorado, United States of America; Faculty of Science, Ain Shams University (ASU), EGYPT

## Abstract

**Background:**

*Rickettsia felis* has recently emerged worldwide as a cause of human illness. Typically causing mild, undifferentiated fever, it has been implicated in several cases of non-fatal neurological disease in Mexico and Sweden. Its distribution and pathogenicity in Southeast Asia is poorly understood.

**Methodology/Principal findings:**

We retroactively tested cerebrospinal fluid (CSF) or sera from 64 adult patients admitted to hospital in North Sulawesi, Indonesia with acute neurological disease. *Rickettsia felis* DNA was identified in the CSF of two fatal cases of meningoencephalitis using multi-locus sequence typing semi-nested PCR followed by Sanger sequencing. DNA from both cases had 100% sequence homologies to the *R*. *felis* reference strain URRWXCal2 for the 17-kDa and *ompB* genes, and 99.91% to *gltA*.

**Conclusion/Significance:**

The identification of *R*. *felis* in the CSF of two fatal cases of meningoencephalitis in Indonesia suggests the distribution and pathogenicity of this emerging vector-borne bacteria might be greater than generally recognized. Typically *Rickettsia* are susceptible to the tetracyclines and greater knowledge of *R*. *felis* endemicity in Indonesia should lead to better management of some acute neurological cases.

## Author summary

*Rickettsia felis* is a flea-borne bacteria that has recently emerged worldwide as a cause of disease. Little is known about its distribution or details of transmission but typically it has been described as causing mild, self-limiting, febrile illness. It has, however, been incriminated in Mexico and Sweden of causing acute neurological disease. We report from North Sulawesi, Indonesia evidence that *R*. *felis* caused or contributed to the death of two adult men admitted to hospital with progressive meningoencephalitis. One case was a farmer and the other was an itinerant urban salesman. DNA unique to *R*. *felis* was identified in the patients’ cerebrospinal fluid using Sanger sequencing techniques. No other potential pathogens were found. These appear to be the first possible cases of fatal *R*. *felis* reported. Because rickettsial infections can usually be quickly, cheaply, and effectively treated with doxycycline and related tetracylines, it is important for clinicians in the region to consider using these antibiotics to treat suspect cases presumptively.

## Introduction

*Rickettsia felis* was first identified as a *R*. *typhi*-like organism in the midgut epithelium of a commercial colony of cat fleas, *Ctenocephalides felis* [1]. Although reactive to *R*. *typhi* antibody, it was subsequently shown to be genomically distinct [2, 3]. It is naturally passaged generationally in fleas and has been found in a variety of other arthropods, including mosquitoes, but the exact mechanisms of transmission, including the possible role of vertebrate reservoirs, are still unclear [4].

Since the description of the first human case in 1994 [5] *R*. *felis* has been increasingly identified worldwide as a cause of disease [6–8]. Typically, it presents as a mild, febrile illness that can be syndromically confused with many other undifferentiated fevers, especially in Africa and Asia, where diagnostic resources are limited. Neurological involvement has been reported in several cases in Mexico [9] and Sweden [10, 11] but not deaths. In this report we describe the identification of *R*. *felis* from the cerebrospinal fluid (CSF) of two fatal cases of acute neurological disease in North Sulawesi, Indonesia.

## Materials and methods

### Ethical approvals

This study was approved by the Medical Research Ethics Committee of R.D. Kandou General Hospital (Ethical Approval No. 066/EC-UPKT/III/2016) and Eijkman Institute for Molecular Biology Research Ethics Commission (Ethical Approval No. 127). All patients or guardians provided written informed consent.

### Case characterization and specimen collection

Testing for *Rickettsia* was done retroactively on specimens collected from cases of acute neurological disease suspected to have infectious etiologies. Criteria for case enrollment and specimen collection have been detailed earlier [12]. Briefly, CSF or serum samples were tested from 64 patients aged >15 years admitted to R. D. Kandou General Hospital, Manado, North Sulawesi, Indonesia—a tertiary referral hospital—during August 2015 to February 2017 with suspected central nervous system (CNS) infection. There were 10 patients with CSF and serum samples, 28 with CSF only, and 26 with serum only. Enrollees had acute onset (≤ 7 days) of fever (axillary temperature >37.5˚C) prior to admission and at least one of the following: altered mental state, seizures, focal neurological findings, or neck stiffness. Human immunodeficiency virus (HIV) status was determined at admission. CSF samples were tested for *Cryptococcus neoformans* (CrAg LFA, IMMY, USA) and Gram stain positive/negative bacteria. In previously reported testing, 14 of 74 cases had molecular or serological evidence of viral infection, including herpes simplex, Epstein-Barr, cytomegalovirus, enterovirus, dengue, and Japanese encephalitis [12].

### Molecular testing and interpretation

Cryopreserved samples of CSF and serum were tested for *Leptospira* spp., *O*. *tsutsugamushi*, and *Rickettsia* spp. DNA was extracted from 38 CSF and 36 serum samples using QIAamp DNA Blood mini kit (QIAGEN, Germany), according to manufacturer instructions. Elution volume was 60 μL. Samples were screened for *Leptospira*, *O*. *tsutsugamushi*, and *Rickettsia* using real-time PCR (qPCR) targeting *lipL32*, 47-kDa and 23S rRNA genes, respectively [13–15]. For *Leptospira* qPCR, a 25 μL qPCR reaction, containing 1x PerfeCTa qPCR ToughMix (Quantabio, USA), 0.9 μM primers, 0.15 μM probe, and 5 μL template DNA. The thermal cycling conditions were incubation for 3 minutes at 95°C, followed by 45 cycles of two-step amplification at 95°C for 15 seconds, and 60°C for 1 minute. For *O*. *tsutsugamushi* and *Rickettsia* qPCR, a 25 μL qPCR reaction, containing 1x PerfeCTa MultiPlex qPCR SuperMix (Quantabio, USA), 0.1 μM primers, 0.2 μM probe, and 5 μL template DNA. The thermal cycling conditions were incubation for 8 minutes at 95°C, followed by 45 cycles of two-step amplification at 95°C for 15 seconds, and 60°C for 1 minute. Fluorescence intensity was read during the 60°C annealing/extension step. A C_t_ value ≤ 40 was considered positive for *Leptospira*, *O*. *tsutsugamushi*, or *Rickettsia*. All qPCR experiments were run on 7500 Fast Real-Time PCR System with the fast ramp speed feature enabled (Applied Biosystems, USA).

*Rickettsia* positive specimens were confirmed using multi-locus sequence typing semi-nested PCR targeting the 17-kDa (R17k31F, R17k469R, R17k2608R), *ompB* (RompB3503F, RompB4293R, RompB4246R), and *gltA* (CS49F, CS1234R, CS1273R) genes [16–18]. No positive control was included in the semi-nested PCR to avoid specimen contamination. First amplification round was performed on a 25 μL reaction containing 1x GoTaq Green Master Mix (Promega, USA), 0.3 μM primers, and 3 μL template DNA. The thermal cycling conditions were 2 minutes at 95°C followed by 40 cycles of amplification at 95°C for 30 seconds, 54°C for 30 seconds, and 72°C for 1.5 minutes, and final extension at 72°C for 5 minutes. Two microliters of the resulting product was used for second round amplification in a 25 μL reaction containing 1x GoTaq Green Master Mix and 0.3 μM primers. The thermal cycling conditions were the same as the first round amplification for each gene, except the amplification cycle were reduced to 35 cycles for the reaction with 17-kDa or *gltA* specific primer, and 25 cycles for the reaction with *ompB* specific primer. Reactions were run on agarose gels and DNA fragments that were excised and purified using QIAquick gel extraction kit according to manufacturer instruction (QIAGEN, Germany).

DNA sequencing was performed using BigDye^™^ Terminator v3.1 cycle sequencing kit, following manufacturer protocol (Applied Biosystems, USA) on 3500XL genetic analyzer (Applied Biosystems, USA), and compared to banked specimens using NCBI BLAST. Sequences were deposited in GenBank: 17-kDa (Accession no. MK820604, MK820607), *ompB* (Accession no. MK820606, MK820609), and *gltA* (Accession no. MK820605, MK820608).

Sequence alignment and phylogenetic analysis were performed using CLC Genomics Workbench version 9.5.2 (QIAGEN, Germany). Phylogenetic tree construction was started using neighbor joining algorithm. Kimura 80 was used as the nucleotide substitution model. Maximum likelihood model with 1,000 bootstrap replications was used to estimate genetic relationships.

## Results

### Molecular testing

**Fig 1:**
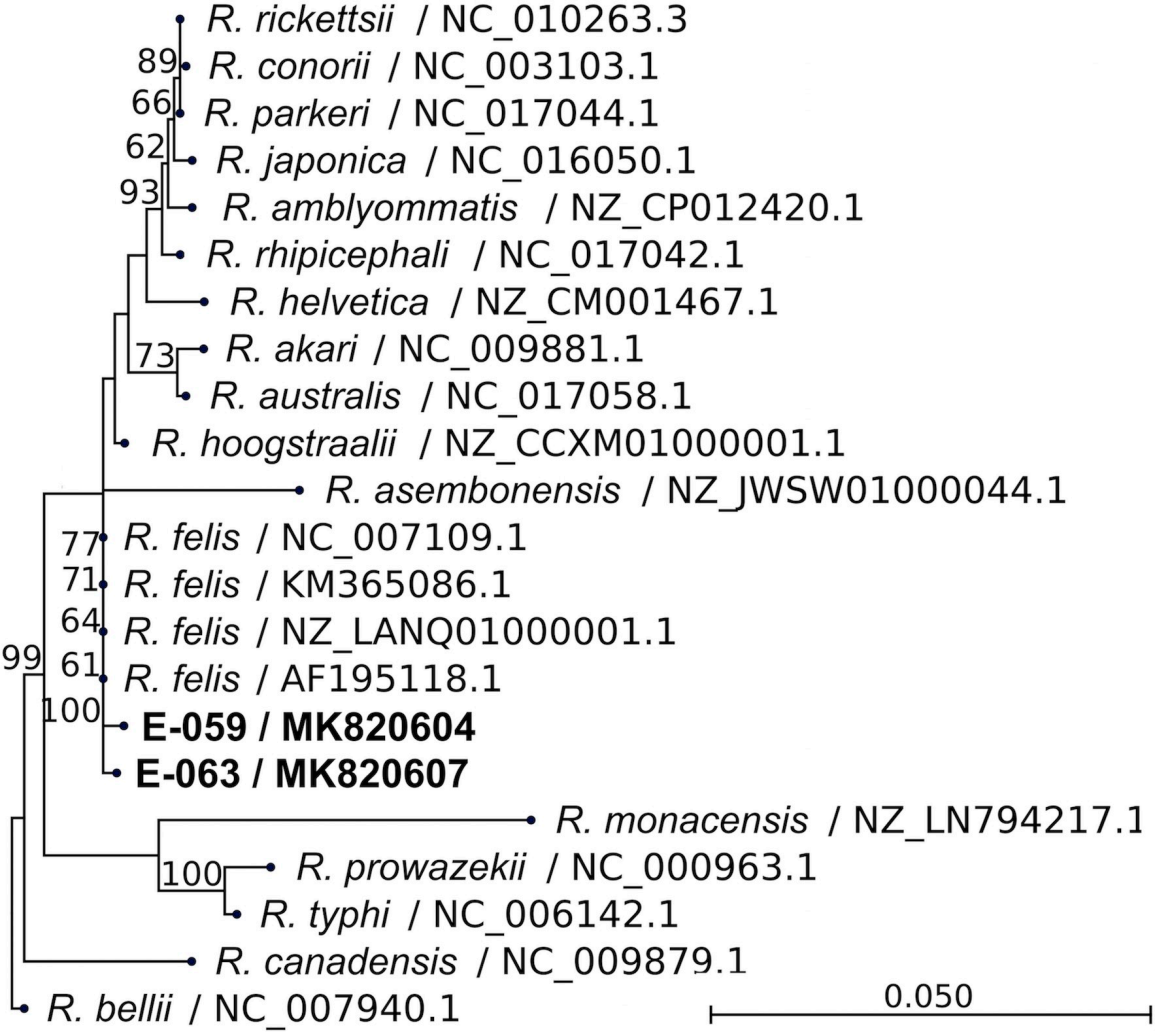
Phylogenetic tree comparing 17-kDa gene fragment of *Rickettsia* from patients to other *Rickettsia* species. Gene fragment size was 381 bp. Bootstrap percentages (≥60%) are shown on internal branches. Label indicates *Rickettsia* species / accession number. The scale bar represents the number of nucleotide substitution per site.

**Fig 2:**
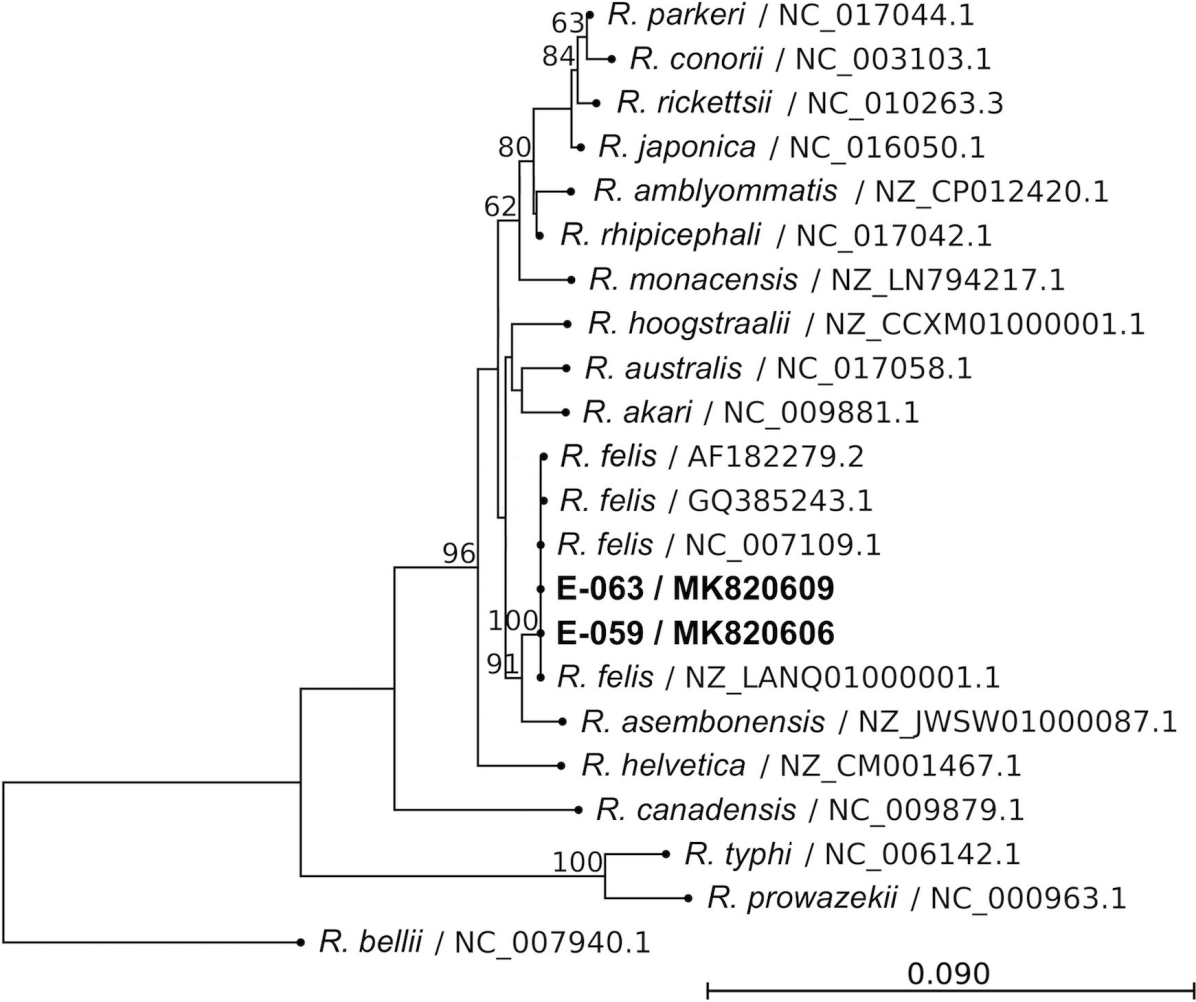
Phylogenetic tree comparing *ompB* gene fragment of *Rickettsia* from patients to other *Rickettsia* species. Gene fragment size was 642 bp. Bootstrap percentages (≥60%) are shown on internal branches. Label indicates *Rickettsia* species / accession number. The scale bar represents the number of nucleotide substitution per site.

**Fig 3:**
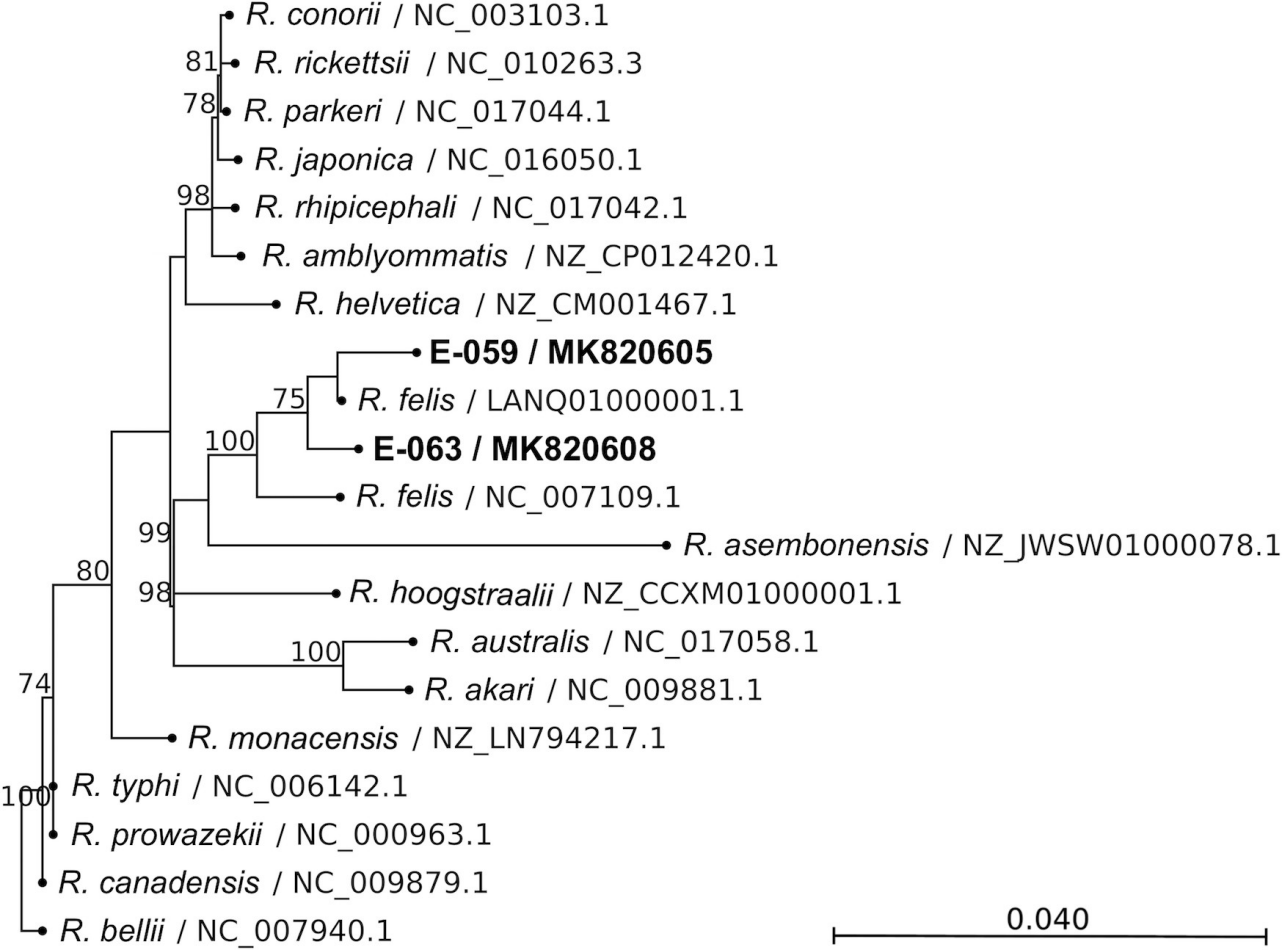
Phylogenetic tree comparing *gltA* gene fragment of *Rickettsia* from patients to other *Rickettsia* species. Gene fragment size was 1,060 bp. Bootstrap percentages (≥60%) are shown on internal branches. Label indicates *Rickettsia* species / accession number. The scale bar represents the number of nucleotide substitution per site.

Thirty-eight CSF and 36 serum samples met volume and condition criteria for PCR testing. All CSF and serum samples were negative for *Leptospira* and *O*. *tsutsugamushi* by qPCR. Two of 38 CSF samples (patients E-059 and E-063) were, however, positive for *Rickettsia* DNA as determined by genus-specific 23S rRNA qPCR. Insufficient sera were available from these two cases for *Rickettsia* PCR. In order to determine the species of *Rickettsia*, semi-nested PCR targeting the 17-kDa, *ompB*, and *gltA* genes in the CSF samples was done. Results were consistent with only *Rickettsia felis* (Table 1). Amplicons were confirmed to be *R*. *felis* by Sanger sequencing (Figs 1–3). Sequence homologies between Case 1 (patient E-059) and Case 2 (patient E-063) of 17-kDa, *ompB*, and *gltA* were, respectively, 100%, 100%, and 99.91%. The 17-kDa and *ompB* sequences were 100% identical to the *R*. *felis* reference strain URRWXCal2 [3], and *gltA* was 99.91% homologous.

**Table 1:** Summary of molecular result of suspected *Rickettsia* infection.

ID	Sample Type	*Orientia tsutsugamushi*	*Rickettsia* spp.	*Rickettsia* multi locus sequence typing genes		
		47-kDa (118 bp)	23S rRNA (111 bp)	17-kDa (434 bp)	*ompB* (733 bp)	*gltA* (1,186 bp)
E-059	CSF	-	+	+ (406 bp)	+ (642 bp)	+ (1,080 bp)
E-063	CSF	-	+	+ (417 bp)	+ (658 bp)	+ (1,060 bp)

### Case histories

Both patients positive for *R*. *felis* DNA were males > 30 years who lived in or near Manado (Coordinates: 1°29’35” N and 124°50’29” E). Detailed clinical manifestations and laboratory data are presented in Table 2. Both presented at admission with acute onset of high-grade fever. Case 1 was an itinerant urban salesman; and Case 2 was a farmer with pre-existing HIV and tuberculosis, *Mycobacterium tuberculosis*, (TB) infections. Case 1 had 4 days history of decreased consciousness preceded by a week of fluctuating bilateral headache. A computerized tomography scan indicated meningeal enhancement and he was found to have CSF mononuclear pleocytosis. Chest x-ray was normal. Case 2 presented initially with normal consciousness and was treated for a lung infection and polyneuropathy; his consciousness began to decrease 2 days post- admission and his fever spiked. This patient was negative for *Cryptococcus* using the lateral flow immunoassay. His chest radiography indicated pleural infiltrations. Computerized tomography scan of his brain was not done. There was no clinical evidence at his admission for either increased intracranial pressure or cranial palsies. Blood cultures were negative for both cases. TB meningitis was initially suspected for both cases but neither CSF was positive by acid-fast bacilli stain and neither responded to presumptive TB treatment. Capacity to culture TB at the hospital was not available. Neither patient was noted on the charts to have skin rash, eschar, or regional lymphadenopathy. No pre-admission histories were available for antibiotic administration or exposure to fleas or ticks. Retroactive testing for virus was negative for both [12].

**Table 2:** Clinical characteristics of patients with suspected *Rickettsia* infection.

Parameter	Patient ID	
	E-059	E-063
**Clinical manifestations and outcome**		
Age (years)	32	38
Sex	Male	Male
High grade fever (duration in days)	Yes (6)	Yes (7)
Day of illness at admission	6	14
Duration of hospital stay (days)	18	7
Headache (duration in days)	Yes (7)	No (0)
Seizure (duration in days)	No (0)	No (0)
Altered consciousness (duration in days)	Yes (4)	Yes (2)
Skin rash	No	No
Meningeal sign	Positive	Negative
Funduscopy	Normal	Normal
Myalgia	No	No
Focal neurologic signs	No	Yes
Glasgow coma scale	12	15
Cognitive impairment	No	No
Hepatosplenomegaly	No	No
Lymphadenopathy	No	No
Respiratory involvement	No	No
HIV-positive status	No	Yes
Tuberculosis-positive status	No	Yes
Fatal outcome / Days after admission	Yes / 18	Yes / 7
**Clinical laboratory investigations**		
Blood		
Hemoglobin (mmol/L)	10.2	5.1
Hematocrit (%)	45.4	19.8
White blood cells (cells/μL)	10,200	3,500
Platelet (counts/μL)	350,000	121,000
Alanine transaminase (IU/L)	7	13
Aspartate transaminase (IU/L)	13	50
Urea (mmol/L)	4.2	2.7
Creatinine (μmol/L)	61.9	44.2
Culture	Negative	Negative
Cerebrospinal fluid		
Total cell count (cells/μL)	122	5
Mononuclear cell proportion (%)	95	10
Polymorphonuclear cell proportion (%)	5	90
Protein (g/L)	2	0.5
Glucose (mmol/L)	1.7	1.3
CSF/serum glucose ratio	<0.4	<0.4
*Cryptococcus neoformans* lateral flow assay	Not done	Negative
Acid-fast bacilli staining	Negative	Negative
Gram staining	Negative	Negative
Chest X-Ray	Normal	Infiltrate
**Neuro-imaging (computerized tomography scan)**		
Meningeal enhancement	Yes	Not done
Focal brain lesions	No	Not done

## Discussion

*Rickettsia felis* is increasingly recognized as a human pathogen, often causing febrile illness clinically indistinguishable from many viral or bacterial infections [6]. In this study, CSF and serum specimens preserved from 64 hospitalized patients with apparent CNS infections were screened for *Rickettsia* DNA; and *R*. *felis* DNA was recovered from the CSF specimens of two fatal cases. Although no other pathogens were detected in the CSF or blood, we cannot definitively incriminate *R*. *felis* as the sole cause of death. Case 2 had a history of treatment for HIV and TB; both cases were presumptively treated for TB after admission. CSF from both were negative by acid-fast bacilli staining; culture and molecular testing for TB were not available at the hospital. Because neither case responded to standard TB therapy for the region, the possibility of an alternate etiology was considered. Hypoglycorrhachia, present in both cases, as well as lymphocytic pleocytosis and increased protein, in Case 1, have been noted in rickettsial infections [19].

*R*. *felis* has been reported as a cause of neurological disease in Mexico [9] and Sweden [10, 11], where it has been identified from CSF. It has also been identified from the CSF of three cases in Laos, each of whom had significant comorbidities, which could have been the principal causes of symptoms [20]. Those cases survived, but fatal neuropathogenicity can be a complication of murine typhus (*R*. *typhi*) and scrub typhus (*O*. *tsutsugamushi*) [19, 21]. Clinically, rickettsial infections can be difficult to distinguish from other causes of acute fever. Platelet counts are often normal [10, 22] and classical signs, such as rash or eschar, are often absent or difficult to detect [11, 21]. Diagnosis is further complicated by the limitations of laboratory assays. Because *Rickettsia* invade endothelial cells, DNA is typically scarce in sera or CSF [23]. Complete pre-admission treatment histories were not known for any of the cases tested.

Although pathogenic *Rickettsia* species are typically vector-borne and *R*. *felis* was originally isolated from cat fleas, its mode of transmission to humans is not certain. It has been found in a variety of arthropod species, including some, like book lice, that are not blood-feeding and others, like mosquitoes, not proven to transmit *Rickettsia* [4, 24]. It is unknown if vertebrate reservoirs contribute to its transmission cycle; the bacterium is transovarially and transstadially maintained in fleas. Taxonomically, *R*. *felis* does not fit neatly into either the traditional TG or SFG and is now considered to be part of a third, transitional group [6–8]. It has a large, complex genome and phylogenetically forms a cluster with a number of recently described “*R*. *felis-*like organisms”, not all of whose members may be pathogens [8]. The Indonesian amplicons were, however, 100% identical to the *R*. *felis* type genome, URRWXCal2, at the 17-kDa (*Rickettsia* genus-specific external membrane) and *ompB* (outer membrane B) genes, and at all but one base at the *gltA* (citrate synthase) gene. The conditions of testing make it unlikely the positives were the result of contamination. Testing was done in a laboratory that had never previously worked with *R*. *felis* or arthropods; no positive controls were present. There has been a report of *R*. *felis* being detected on the skin of healthy West Africans [25]; regardless of how this could be, our procedures for lumbar puncture and testing would make skin contamination from the patients or medical personnel highly unlikely.

Until more is known about the epidemiology of *R*. *felis*, little useful can be surmised about relative risk. Scrub typhus, murine typhus, and *R*. *felis* have been described in Indonesia, mostly from vertebrates and arthropods [26]. One of the positive Indonesian cases was a farmer and the other dwelt in the city; both are environments that could be conducive to flea-borne transmission. Rickettsial disease, in general, appears to be substantially underreported in Southeast Asia [21, 27] and the relative importance of *R*. *felis*, specifically, is unknown. In localities where incidence is suspected to be high the possibility of rickettsial infection in acute neurological disease should be included in clinical evaluations, especially considering the benefits of early empirical treatment. Until capacity for accurate diagnostic testing is much more widespread, however, patient care in the endemic areas will be seriously hindered.
